# A Rapid Systematic Review of Public Responses to Health Messages Encouraging Vaccination against Infectious Diseases in a Pandemic or Epidemic

**DOI:** 10.3390/vaccines9020072

**Published:** 2021-01-20

**Authors:** Sadie Lawes-Wickwar, Daniela Ghio, Mei Yee Tang, Chris Keyworth, Sabina Stanescu, Juliette Westbrook, Elizabeth Jenkinson, Angelos P. Kassianos, Daniel Scanlan, Natalie Garnett, Lynn Laidlaw, Neil Howlett, Natalie Carr, Natalia Stanulewicz, Ella Guest, Daniella Watson, Lisa Sutherland, Lucie Byrne-Davis, Angel Chater, Jo Hart, Christopher J. Armitage, Gillian W. Shorter, Vivien Swanson, Tracy Epton

**Affiliations:** 1 Department of Primary Care and Population Health, University College London, London NW3 2PF, UK; 2 Department of Psychology, Faculty of Health and Society, University of Salford, Manchester M6 6PU, UK; d.ghio@salford.ac.uk; 3 Behavioural Science Policy Research Unit, Population Health Sciences, Newcastle University, Newcastle upon Tyne NE2 4AX, UK; meiyee.tang@newcastle.ac.uk; 4 Manchester Centre for Health Psychology, University of Manchester, Manchester M13 9PL, UK; chris.keyworth@manchester.ac.uk (C.K.); lucie.byrne-davis@manchester.ac.uk (L.B.-D.); jo.hart@manchester.ac.uk (J.H.); chris.armitage@manchester.ac.uk (C.J.A.); tracy.epton@manchester.ac.uk (T.E.); 5 School of Psychology, University of Southampton, Southampton SO17 1BJ, UK; s.stanescu@soton.ac.uk; 6 Department of Psychology, University of Bath, Bath BA2 7AY, UK; jsw46@bath.ac.uk; 7 Department of Health and Social Sciences, University of West England, Bristol BS16 1QY, UK; elizabeth2.jenkinson@uwe.ac.uk (E.J.); natalie2.Garnett@live.uwe.ac.uk (N.G.); ella.guest@uwe.ac.uk (E.G.); 8 Department of Applied Health Research, University College London, London WC1E 6BT, UK; angelos.kassianos@ucl.ac.uk; 9 Department of Communication, Policy, and Research, Education Support, London N5 1EW, UK; Daniel.scanlan00@gmail.com; 10 Public Contributor, Health Psychology Exchange Patient and Public Involvement (PPI) Group, UK; lynnlaidlaw0@gmail.com; 11 Department of Psychology, Sports, and Geography, School of Life and Medical Sciences, University of Hertfordshire, College Lane, Hertfordshire AL10 9AB, UK; n.howlett@herts.ac.uk; 12 Faculty of Health, Psychology, and Social Care, Manchester Metropolitan University, Manchester M15 6BH, UK; nataliecarr_1@hotmail.co.uk; 13 Faculty of Health and Life Sciences, School of Applied Social Sciences, De Montfort University, Leicester LE1 9BH, UK; natalia.stanulewicz@dmu.ac.uk; 14 Global Health Research Institute, Human Development and Health, Faculty of Medicine, University of Southampton, Southampton SO16 6YD, UK; d.watson@soton.ac.uk; 15 Behavioural Insight, Edinburgh EH9 3EY, UK; lisaasutherland@googlemail.com; 16 Division of Medical Education, University of Manchester, Manchester M13 9PT, UK; 17 Centre for Health, Wellbeing and Behaviour Change, University of Bedfordshire, Bedford, Bedfordshire MK41 9EA, UK; angel.chater@beds.ac.uk; 18 NIHR Manchester Biomedical Research Centre, Manchester University NHS Foundation Trust, Manchester M13 9WL, UK; 19 Manchester Academic Health Science Centre, Health Innovation Manchester, Manchester M13 9NQ, UK; 20 Centre for Improving Health Related Quality of Life, Queen’s University Belfast, Belfast BT7 1NN, UK; g.shorter@qub.ac.uk; 21 Department of Psychology, University of Stirling, Stirling FK9 4LA, UK; vivien.swanson@stir.ac.uk

**Keywords:** public health messaging, vaccine uptake, vaccine hesitancy, pandemics, epidemics, systematic review

## Abstract

Public health teams need to understand how the public responds to vaccination messages in a pandemic or epidemic to inform successful campaigns encouraging the uptake of new vaccines as they become available. A rapid systematic review was performed by searching PsycINFO, MEDLINE, healthevidence.org, OSF Preprints and PsyArXiv Preprints in May 2020 for studies including at least one health message promoting vaccine uptake of airborne-, droplet- and fomite-spread viruses. Included studies were assessed for quality using the Mixed Methods Appraisal Tool (MMAT) or the Assessment of Multiple Systematic Reviews (AMSTAR), and for patient and public involvement (PPI) in the research. Thirty-five articles were included. Most reported messages for seasonal influenza (*n* = 11; 31%) or H1N1 (*n* = 11; 31%). Evidence from moderate to high quality studies for improving vaccine uptake included providing information about virus risks and vaccination safety, as well as addressing vaccine misunderstandings, offering vaccination reminders, including vaccination clinic details, and delivering mixed media campaigns across hospitals or communities. Behavioural influences (beliefs and intentions) were improved when: shorter, risk-reducing or relative risk framing messages were used; the benefits of vaccination to society were emphasised; and beliefs about capability and concerns among target populations (e.g., vaccine safety) were addressed. Clear, credible, messages in a language target groups can understand were associated with higher acceptability. Two studies (6%) described PPI in the research process. Future campaigns should consider the beliefs and information needs of target populations in their design, including ensuring that vaccine eligibility and availability is clear, and messages are accessible. More high quality research is needed to demonstrate the effects of messaging interventions on actual vaccine uptake.

## 1. Introduction

Scientists have made significant, rapid breakthroughs to protect communities against the novel SARS-CoV-2 virus and several vaccines have been approved globally [1,2,3,4]. Vaccination reduces the burden of infectious diseases, which can be eliminated locally if enough of the population takes up the vaccine [5], with 80% of healthy people and 90% of high-risk individuals reportedly required to establish herd immunity against influenza [6]. However, there is concern that not enough people will take up vaccines against SARS-CoV-2 once they become available. Global surveys of adults found willingness to have a vaccination (i.e., those who agreed or strongly agreed that they would get a SARS-CoV-2 vaccine if it were available) was 71.5% in June [7], 74.5% in August, 72.4% in October, and 65.8% in December 2020 [8]. Among adults at increased risk of developing severe COVID-19 (including older people, people with existing long-term conditions, and pregnant women), 80% were willing to accept a new vaccine when surveyed between April and June 2020 [9]. Even if willingness translates perfectly into vaccine uptake, it is still likely that vaccine hesitancy will impact the vaccination effort against SARS-CoV-2 among at-risk groups. There is some evidence that this hesitancy is increasing in healthy adults in the majority of countries, with vaccine hesitancy above 50% in France and Russia [8].

Research has shown a variety of psychological factors are associated with vaccine hesitancy. Beliefs can be held on risk of infection, severity of the public health issue, severity of personal consequences due to illness, the consequences of vaccination [10,11], and the effectiveness of vaccines [11,12]. Reasons for hesitancy towards having a vaccine against SARS-CoV-2 include a lack of understanding about vaccine eligibility, worry about side effects, beliefs that the vaccination is not effective, perceptions of not being at sufficient risk from SARS-CoV-2, being against vaccines in principle, and not having the time [8,10].

Early findings also demonstrate variation in hesitancy among sub-groups within the population. Respondents who were younger, from Black, Asian, and minority ethnic (BAME) backgrounds, or had lower education levels, were significantly less willing to be vaccinated [9,13]. Smokers and people who had previously contracted SARS-CoV-2 have also been found to be less willing to be vaccinated [9]. This suggests that people who are hesitant are likely to hold different beliefs and values, and any future efforts to encourage vaccination should account for this variation.

Furthermore, intentions to receive a vaccination, and vaccination uptake, have been found to vary over the different phases of a pandemic. During the H1N1 pandemic, studies highlighted a declining trend, with intention decreasing post-pandemic when a vaccine became available [11]. A seasonal influenza vaccine is offered each year in the UK to individuals at risk of poor influenza outcomes, with uptake among adults over 65 between 71% and 75% [14], but uptake among other clinical risk groups under 65 is lower and trending downwards from 48% in 2018–2019 to 45% in 2019–2020 [15]. This suggests that there may be changes in the intentions and actions of individuals over time that may be influenced by reduced risk perception as a pandemic or epidemic becomes more controlled and treatments are improved. This may well have been the case for H1N1 where the vaccine became available post-pandemic once the virus had run its course [11].

Determining the success of previous health campaigns relating to pandemics and epidemics can inform future communication strategies for promoting vaccine uptake. The best evidence for a successful vaccination campaign is if the messaging affects uptake. Changes in uptake are mediated by psychological processes, and public health campaigns should formally consider variables such as intentions, beliefs, and gaps in understanding about vaccines and how they work. Campaigns should also consider the public’s changing information needs during the various phases of a pandemic or epidemic as well as the needs of particular groups. One way to improve public health messages is to include target populations in their design and dissemination. A recent comprehensive review of public health messaging recommended engaging communities in the development of public health messages [16]. By involving key stakeholders in the design of public health messaging, materials will be co-created with the understanding of those we wish to engage in target behaviours. The aim of the present review therefore was to identify and synthesise evidence relating to effective messaging for encouraging vaccination in order to prevent virus transmission during pandemics or epidemics. The degree to which the public have been involved in public health messages included in this review will also be determined.

## 2. Materials & Methods

### 2.1. Protocol

The protocol for a broader systematic review of public health messaging was amended on 6th August 2020 to include the present review of vaccine messaging: PROSPERO Ref. CRD42020188704.

### 2.2. Search Strategy and Selection Criteria

Searches for published and unpublished studies were performed in May 2020 using Ovid PsycINFO, Ovid MEDLINE and healthevidence.org, and OSF Preprints and PsyArXiv Preprints, respectively. All research designs were considered for inclusion. The search strategy was developed and conducted for a broader systematic review of public responses to public health messaging by the same authors and is reported elsewhere [16]. Retrieved references were exported into Rayyan [17]. We conducted a keyword search including “vaccine”, “vaccines” and “vaccination” of all studies identified as eligible for full text screening [16]. All studies including these terms in the title or abstract were screened at full text by two of nine of the authors (S.L.-W., D.G., M.Y.T., J.W., S.S., E.J., N.G., D.S. & A.P.K.) and any disagreements were reviewed by a third author until consensus was reached. Reference lists of eligible studies were hand searched only if the article mentioned potentially relevant additional studies, due to the rapid nature of this review. Non-English language articles and dissertations were also excluded due to time restrictions.

Studies were included if they tested the impact of at least one type of public health message (e.g., television broadcasts, websites, text alerts) on vaccination-related behaviours or psychological variables with adults, and included viruses spread from human-to-human with primary transmission being airborne, droplet and fomite (touch) contact. Studies which focused on sexually transmitted infections, for example HIV, were excluded as they were considered to be significantly different from those of interest for this review. Studies already contained within eligible systematic reviews were not included as individual primary studies to avoid duplication of data and over-emphasising evidence from a single study.

### 2.3. Data Extraction

A data extraction form was developed by the authors based on the SPICE criteria (Setting, Perspective, Phenomena of Interest, Comparison, Evaluation, Time Scope) [18].

### 2.4. Risk of Bias Assessment

Quality assessment checks were performed independently by six of the review authors (S.L.-W., E.J., L.B.D., D.S., D.G. & M.Y.T.) for all eligible articles included in the review. Double quality assessment was not performed due to time restrictions. The Mixed Methods Appraisal Tool (MMAT [19]) was used to review the quality of primary studies of any design. The Assessment of Multiple Systematic Reviews (AMSTAR [20]) was used to review the quality of reviews and systematic reviews. Therefore, each included article received one quality score. Low quality was categorised as 0–1 on MMAT or 0–3 on AMSTAR; moderate quality was categorised as 2–3 on MMAT and 4–6 on AMSTAR; and high quality was categorised as 4–5 on MMAT and 7–10 on AMSTAR.

### 2.5. Patient and Public Involvement (PPI)

We performed an assessment of the involvement of patients or the public in the final included studies. Two of the authors (T.E. & L.L.) developed a PPI Checklist, which was based on a study investigating the reporting of PPI within research articles [21]. The checklist was used to rate the type and extent of PPI that has been reported in studies, for example being involved in the study steering group by responding “No”, “Yes”, or “Unclear” for each type of PPI. Two additional authors (D.G. & S.L.-W.) reviewed the draft version of the PPI Checklist and all four authors agreed on the final version (Supplementary Table S1).

## 3. Results

### 3.1. Study Selection

**Figure 1:**
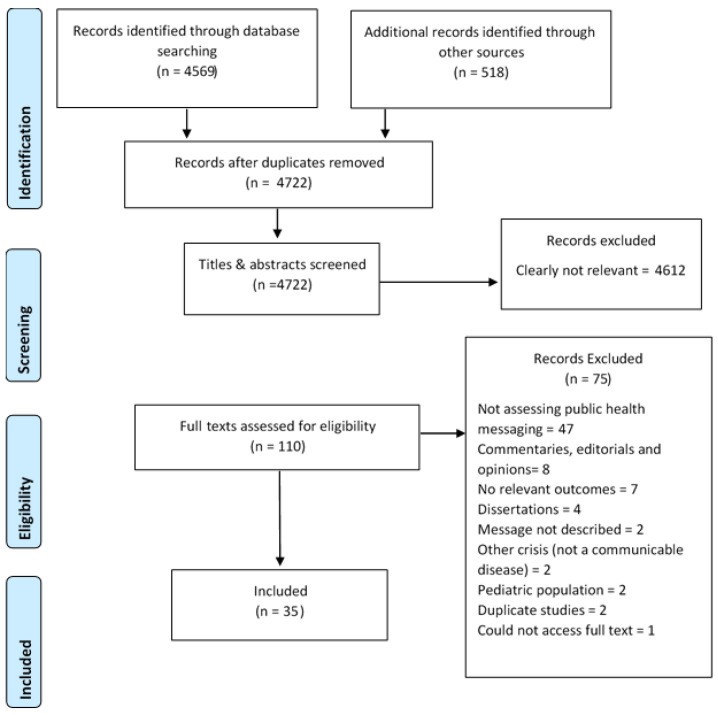
PRISMA Flow Chart.

The full texts of 110 articles identified from the broader review of public health messaging [16] were screened for eligibility. A total of 35 studies were included in the review (Figure 1), the majority being primary studies (*n* = 30), with a small number of systematic reviews (*n* = 3) and two editorial reviews (*n* = 2).

### 3.2. Study Characteristics

Characteristics of the articles included in the review and the types of intervention evaluated in included studies are presented in Supplementary Table S2. Messages included emails, letters, leaflets, text messages, websites, television broadcasts, newspaper articles, and mass media campaigns and encouraged vaccination for: seasonal influenza (*n* = 11) [22,23,24,25,26,27,28,29,30,31,32], H1N1 influenza (*n* = 11) [33,34,35,36,37,38,39,40,41,42,43], measles, mumps and rubella (*n* = 1) [44] and pneumococcal infection (*n* = 1) [45]. Two studies reported potential future vaccination for avian influenza (*n* = 1) [46] and Ebola (*n* = 1) [47]. A further nine studies reported messages for vaccines for influenza that were not specified (*n* = 4) [48,49,50,51] and hypothetical influenza scenarios (*n* = 5) [52,53,54,55,56]. Studies were conducted in the United States (US) [22,23,24,27,28,32,37,38,39,40,44,45,46,48,51,53,54], Singapore [25], UK [26,29,43,52,55], Italy [30,42], Australia [31,36], Hong Kong [32], China [33], Taiwan [34,41], Canada [35,56], Germany [49], Thailand [50], and Switzerland [47]. Study populations included college or university students and/or staff (*n* = 10) [22,27,28,32,34,39,43,47,49,54], general public (*n* = 9) [25,33,42,45,46,52,53,55,56] adults over 50 years (*n* = 4) [24,30,40,48], pregnant women (n = 3) [23,38,51], hospital attendees (including non-clinical staff) (*n* = 2) [36,41], adults with long-term conditions or unspecified “high risk” (*n* = 3) [26,31,50], healthcare workers (*n* = 1) [36] and Aboriginal First Nations and Metis adults (*n* = 1) [35]. A population of interest was not specified for three reviews [29,37,44].

### 3.3. Risk of Bias

Half of the studies (*n* = 18; 50%) scored highly on their respective quality assessment tools [23,26,29,30,31,35,36,37,38,40,43,48,52,53,54,55,56] (Table 1). Three studies and two editorial reviews were found to be of low quality [32,41,44,49,51] while the remaining were of moderate quality.

### 3.4. Results of Individual Studies

A summary of the main results is reported in Table 1.

### 3.5. Synthesis of Results

#### 3.5.1. Evidence of Impact of Messaging on Behaviour

A total of 12 out of 35 included articles reported vaccination uptake among their populations of interest. These were for the seasonal influenza [22,23,26,29,31], H1N1 [33,36,37,42], unspecified influenza [50,51], and pneumococcal vaccines [45]. Eight studies described successful vaccine promotion campaigns for increasing uptake. These included community- [29,45] or hospital-wide [29] mixed media messages, text message prompts sent from local physician clinics to adults from high risk groups [23,31,51], text prompts with information about virus prevention and addressing misunderstandings about vaccination [33], the inclusion of a map with vaccination clinic locations in an email [22], and ensuring messages were credible, clear and provided honest information about pandemic influenza vaccination [37]. Studies showing no impact of messages on vaccination included leaflets providing information about influenza and the benefits of vaccination [50], a text message reminder prompt from a local practice [26] and an educational TV campaign encouraging a range of preventative behaviours including vaccination [42]. One cross-sectional study did not measure vaccine uptake robustly enough to make conclusions about whether mixed media messages had an impact on this behavioural outcome [36].

#### 3.5.2. Evidence of Impact of Messaging on Behavioural Influences

##### Intentions or Willingness to Take up Vaccination

A total of 18 out of the final 35 studies reported either intentions, or willingness, of individuals towards taking up vaccination for seasonal influenza [25,27,28,29,30,32], H1N1 [34,36,38,40], unspecified influenza [50], Ebola [47], MMR [44], avian influenza [46] and in a hypothetical influenza scenario [52,53,54,55,56]. Eleven studies described successful vaccine promotion messages for improving either intentions or willingness, including framing losses when the message was collectivist [32,40], and when text was used [28,34], framing gains by emphasising benefits to society [44,46], using formal (rather than colloquial) language [25] and shorter messages [52], leaflets including information about virus susceptibility and severity and benefits of vaccination [50], presenting risks in a socially and personally relevant way [56], media multitasking while viewing health websites [54], and presenting messages on white backgrounds with red text [34]. Interventions showing no impact of messages on intentions or willingness included using personal stories in messages [30] and messages including basic risk information alone [27]. Emphasising uncertainty in messages [53] had a negative impact on willingness to be vaccinated in a hypothetical influenza scenario.

##### Beliefs and Attitudes about Vaccines and Vaccination

A total of 15 of the final 35 studies reported the attitudes (i.e., the positive and negative evaluations of the vaccines) and beliefs (e.g., beliefs about capability to take up vaccines, beliefs about the consequences of having a vaccine) of the general public or specific population groups towards vaccination for seasonal influenza [27,28,30,32,36], Ebola virus [47], H1N1 [40,43], and unspecified [48,49,50] or hypothetical [52,53,55,56] influenza viruses, and the impact of messages on these beliefs. Messages improving attitudes towards vaccination included loss framed messages with collectivist appeals [32] when presented as text (rather than images) [28], and gain framed messages including images [28]. Successful messages for improving perceived effectiveness or benefits of vaccines emphasised reduction in risks [52] and used relative risk framing [56]. Leaflets that were personally relevant and emphasised susceptibility and severity of viruses and the benefits of vaccination increased beliefs about capability to get vaccinated [50], as did narrative messages including personal accounts of people who took up a vaccine [30]. Campaigns which influenced negative beliefs about vaccination included messages not providing honest safety information about vaccines [55], messages using “fear appeals” including images of children with MMR [44] and over-emphasising the dangers of viruses [47], and emphasising uncertainty [53]. Three studies also reported that providing official information sources [49], emphasising the safety and effectiveness of vaccines [48], and providing basic information about the risk of vaccines [27] were also found to have a negative impact on beliefs about vaccine effectiveness.

#### 3.5.3. Evidence on Information Needs

A total of 12 of the final 35 studies reported acceptability to the general public or specific population groups of messages, and/or on levels of knowledge or understanding of the information shared in messages. These studies were conducted in the contexts of seasonal influenza [24,28,30,32], H1N1 [34,35,38,39], hypothetical influenza [52,55], and unspecified influenza [48,50]. Messages found acceptable by target audiences include factual, risk-reducing messages [55], narrative messages [30] gain-framed messages [32], loss-framed messages [34], and risk-reducing messages [52]. Less acceptable messages included health-enhancing messages [28], and those eliciting anticipated regret [28]. Two moderate quality studies found that vaccination campaigns improved knowledge about side effects [24,50]. When only facts were used, this did not improve information recall over the use of facts alongside myths, or when facts, myths and refutations were used [24]. Both leaflets used in a study by Payaprom et al. [50] also improved knowledge of side effects, and were similar in their inclusion of details about influenza and the benefits of vaccination.

Information needs of target populations highlighted by studies included gaps in understanding of how long it takes to build immunity following vaccination [48], whether vaccines for one virus can offer protection against another [36], and unfamiliarity with vaccine-related terminology [38]. When health information was presented through narratives whereby the target population was the centre of the story, these messages were better understood than didactic messages that aimed to instruct [30].

### 3.6. Patient and Public Involvement (PPI)

Only two out of 35 articles described the involvement of patients or the public in the research process. One study included the public in the intervention design and in the steering group [45]. A further study involved the public in identifying priorities and in the design [35]. No studies involved patient or public involvement groups in the analysis or interpretation of their findings.

## 4. Discussion

### 4.1. Summary of Evidence

The aim of this rapid systematic review was to identify and synthesise evidence relating to effective messaging on vaccination-related behavioural or psychological variables in a pandemic or epidemic. This review identified a variety of messages reported by 35 articles used to encourage vaccine uptake. There is evidence among moderate to high quality studies of suitable message content and targeting for improving vaccine uptake. There was also evidence from moderate to high quality studies of suitable message formatting, framing and content to support vaccination beliefs and intentions, message comprehension and acceptability. These findings are supported by previous evidence related to other public health campaigns identified in a broader systematic review of messaging not specific to vaccine uptake [16] (Table 2).

### 4.2. Improvements to Messaging

Our findings indicate there is room for improvement in future vaccination campaigns during pandemics and epidemics, not only from the evidence for successful campaigns, but also where our review has highlighted inconsistencies. Our review found how messages are framed, in terms of the losses of non-vaccination and gains associated with vaccination, can have an impact on intentions to vaccinate. However, there were mixed findings on the way that potential gains and losses should be framed to improve vaccination beliefs and intentions. The evidence was generally of poor quality [28,32,40] and as such we identified no good quality evidence that loss-framed messages were more effective than gain-framed messages for increasing intentions. Furthermore, whilst one moderate quality study reported formal rather than colloquial language improved intentions [25], this evidence is limited and it may be useful to consider language with caution. Studies measuring message comprehension have identified specific information needs of target populations, including the need to consider literacy and unfamiliarity with scientific terminology [38,53]. It is important that public health teams ensure messages are clear, use an appropriate message frame, and are delivered in language target populations can understand. This can be achieved by co-designing messages alongside the communities teams are targeting in messages.

Risk information, and information about vaccine efficacy and benefits, can influence intentions or willingness to vaccinate but may need to be presented in a particular way. Relative risk information, for example expressing a risk reduction from 4% to 2% as ‘‘reduced by 50%”, was more effective at increasing willingness to vaccinate against a hypothetical influenza than absolute risk information, for example expressing the same risk as “2% lower” [56]. The addition of risk information improved willingness to vaccinate when it was presented in a socially and personally relevant way in another study in the same high quality systematic review [56,61]. However, one moderate quality study found that the inclusion of risk information decreased intentions for season influenza vaccination uptake [27]. Risks of inaction, when presented as images or “dramatic” narratives in messages, can have a negative impact on beliefs about vaccines, as found by one low quality review of messages encouraging MMR vaccination where parents were more likely to be concerned about vaccine side effects [44].

Evidence was also mixed from trials of text messages encouraging vaccine uptake, where uptake reportedly improved in four studies (ranging in quality) [23,31,33,51], but no effects were demonstrated in a fifth, high quality, study [26]. Notably, text messages were found in one review to be especially effective in certain populations, who may not represent more disadvantaged groups or people at high risk of severe disease [23]. Text messages were found especially effective in white people, aged 25–49, college educated, married, working, living at or above poverty level and having high risk conditions [23]. This suggests that public health teams should review the delivery preferences of target audiences and ensure intervention delivery methods (e.g., text message) are both accessible and acceptable for target groups.

Messages that had negative impacts on the beliefs and intentions of target populations include health-enhancing messages, which were considered lacking in honesty about the potential harms of vaccination in two high quality studies [52,55]. This is in line with research demonstrating that trust reduces if information in public health messages is perceived to have been exaggerated [16]. Two high quality studies found factual, risk-reducing messages were more effective than health-enhancing messages in encouraging beliefs that vaccination was more beneficial [52], and on perceptions that the message was more convincing and credible, than health-enhancing messages [55]. This highlights the importance of offering credible, honest, clear information in messages encouraging vaccination during health crises. Credibility and believability can increase the acceptability of messages. Public health authorities should therefore consider using a credible source in messaging, as supported by recent British Psychological Society (BPS) guidance for public health messaging during pandemics and epidemics [62].

This review also identified concern among populations about the risks of vaccines prompted by messages, which impacted on beliefs about vaccine effectiveness, including concerns about the consequences of vaccination (e.g., side effects), vaccine safety, and the speed vaccines have been developed to manage global pandemics. Similar concerns have been expressed by the public during the expedited development of vaccines for SARS-CoV-2. Communication about rapid development risks can damage public confidence in the vaccines [63]. This suggests that encouraging vaccination through messages may involve taking a more balanced approach in order to provide appropriate information to resolve the concerns of target populations. A recent systematic review of studies from the H1N1 pandemic found reporting the threat of a virus honestly, presenting both known and unknown factors, can improve the population’s perceptions and trust during times of public health crisis [37]. This is consistent with research specific to SARS-CoV-2 which found individuals reporting higher levels of trust in information from government sources were more likely to accept a new vaccine for SARS-CoV-2 [10]. Building trust within communities and ensuring messages come from trusted authorities have been identified as key strategies for any effort to support vaccination uptake in a recent report on behavioural considerations for acceptance and uptake of SARS-CoV-2 vaccines from the World Health Organization (WHO) [64].

The potential for public health messages to cause confusion among the public is an important finding and indicates that messages need to be well designed and timed. For example, confusion about whether or not the seasonal influenza vaccine was protective against H1N1 was perhaps unsurprising given government campaigns relating to the H1N1 pandemic also encouraged the public to be immunised against seasonal influenza [36]. Messages should therefore consider the impact of encouraging vaccination for multiple viruses, which may have differing eligibility criteria. Indeed, a lack of understanding about eligibility for vaccines in general was found in a recent survey [9]. These issues should be clarified in public messages, including clearly communicating the population(s) eligible for vaccines. For example, this review included the impact of messages encouraging influenza vaccine uptake among pregnant women, with hesitancy identified during the H1N1 pandemic, and which was commonly related to beliefs about the vaccine being new and the consequences of vaccination (e.g., fearing side effects) [38]. However, pregnant women are currently advised not to take up vaccines approved in the UK for SARS-CoV-2 [65]. Messages should be tailored when new information becomes available as the pandemic or epidemic evolves [11,14,65].

This review identified evidence highlighting the importance of consulting groups at higher risk of contracting viruses when developing messages, so campaigns are sensitive to their needs and are not perceived to be discriminatory [35]. Arguably, the involvement of target populations should be central to future campaigns during pandemics or epidemics, to ensure the concerns of specific populations are relevant and are addressed in an appropriate way [16]. Particularly in the context of communicating risks within public health messages, the WHO recommends including the community in planning, information dissemination, and relationship building during public health crises to improve the public’s preparedness and responses [66]. Evidence suggests that the public pay more attention to messages if the community are involved in their development [16]. Only two studies included in this review mentioned including patients or the public in the research team. Future campaigns should therefore involve target audiences at the centre of their design and evaluation.

A key outcome to evaluate the effectiveness of a vaccine message is to measure performance of the actual behaviour (i.e., getting the vaccine). Despite this, only 12 out of the final 35 included articles reporting vaccination uptake among their populations of interest. It is important to note that vaccination willingness or intentions, no matter how strong, may have little impact on behaviour. Willingness in particular lacks premeditation and is more weakly related to behaviour than intentions [67]. Only one study in this review explored whether intentions translated into vaccination uptake and found no influence [22]. This can be explained by the intention–behaviour gap which suggests intentions can be useful but are generally insufficient predictors of behaviour [68,69]. The intention–behaviour gap describes the failure to translate what we intend to do into action and has been studied in influenza vaccine hesitancy [70]. This suggests fewer people reporting intentions to receive a vaccine would be likely to go on to be vaccinated and has implications for medical practice, particularly where vaccination involves adhering to two doses, such as in vaccines currently being approved for SARS-CoV-2. Intentions have also been found to change or waver over time, particularly during a pandemic or epidemic with no clear endpoint. Unstable intentions have been found to weaken the intention–behaviour relationship in the context of influenza vaccination uptake [71]. This study highlights how messaging can be used to stabilise intentions and strengthen the likelihood of following recommended behaviours. However, more high quality research evaluating the impact of vaccine messaging on behavioural outcomes is needed for firm conclusions to be made.

This review has highlighted that more evaluations of public health campaigns encouraging vaccination during pandemics or epidemics are needed. Notably only three RCTs of interventions were identified [26,31,33], with this review consisting mainly of cross-sectional studies. All three RCTs compared vaccination text-messaging interventions to a standard care group. The control conditions varied and in settings where texts were delivered by general practices it is possible that control participants were also exposed to local public health campaigns. This highlights the need for more RCTs testing different types of messages over time, and capturing behavioural outcomes appropriately, as a pandemic or epidemic situation evolves. More research is also needed to establish whether the medium through which a message is delivered, e.g., text message, affects vaccine uptake. Evaluation plans should be embedded into the development of campaigns encouraging vaccination to answer some of these remaining questions. Furthermore, this review identified only a small number of European studies, suggesting more studies are needed to establish the impact of efforts to encourage vaccination during pandemics and epidemics across Europe.

### 4.3. Study Limitations

The studies included in this review were found to vary in quality. It is important to note the inclusion of experimental studies testing messages for hypothetical pandemic or epidemic scenarios (*n* = 5; 14%) where responses might not reflect those given by individuals living through a global or local health crisis. Where the type of influenza virus in a further four studies was not specified, it is possible these scenarios too were hypothetical and the results from these studies may not be reflective of the reactions of the public to a genuine virus. Furthermore, almost a third of included studies recruited university student samples (*n* = 10; 29%), the limitation of this being a disproportionate focus on the views and experiences of young adults, as compared to other groups (e.g., high risk groups) who are notably under-represented in studies testing vaccination messages identified in the present review. Meta-analysis was not possible in this review due to the heterogeneity of outcomes and outcome measures used by public health messaging studies. This highlights the need for a core outcome set (COS) in moving evaluation research forward when measuring vaccine hesitancy and the impact of public health messages.

This was a rapid systematic review, conducted under time constraints in order to be relevant and effective for the current pandemic. We used a small selection of vaccination-related terms to search for relevant studies among a pool of studies identified using non-vaccination terminology. However, the authors also added vaccination-related terms to the original search for studies related to public health messaging (reported elsewhere [16]), and checked references lists of key articles, which identified a similar number of additional articles, so while we might have omitted some relevant studies, this is unlikely to have been a significant number. Rapid review methods also included omitting non-English language articles and dissertations, and it is possible relevant research was excluded.

## 5. Conclusions

The responses of the public to previous messages encouraging vaccines for epidemic or pandemic viruses could inform future campaigns for novel viruses such as SARS-CoV-2. Messages could be improved by ensuring they address the information needs of target populations, use credible sources, are personally relevant, shorter, and are honest about what is known about vaccines without over-emphasising the health benefits of vaccination. Vaccine eligibility should be clear, which may involve tailoring messages as new information becomes available. Health authorities designing campaigns should review the delivery preferences of target populations to ensure messages are accessible and acceptable. Future public health campaigns should involve members of the public, and in particular people with lived experience of being at high risk of epidemic or pandemic viruses, in their design and evaluation. Overall, quality of the studies included in this review was moderate to high and the results of low-quality studies should be viewed with caution. There is a need for more rigorous evaluations of public health campaigns encouraging vaccine uptake, measuring behavioural outcomes.

## Figures and Tables

**Table 1:** Results of the included studies.

Authors, Year	Attitudes towards the Vaccine Message	Attitudes towards Vaccination	Beliefs about Effectiveness of Vaccine	Beliefs about Capability of being Vaccinated	Intentions to be Vaccinated	Vaccination Uptake	Other	Mixed Methods Appraisal Took (MMAT)/Assessment of Multiple Systematic Reviews (AMSTAR) Score
Baskin, 2018 [22]						The only significant variable was the map condition, which increased the probability of getting vaccinated overall by 2%.		2
Bushar et al., 2017 [23]						Influenza vaccination coverage for women who received text messages was 81.3% compared with 47.1% for non-text message control group		5
Cameron et al., 2013 [24]							The Centre for Disease Control & Prevention (CDC) control message was more effective in increasing participant knowledge than the facts only message. Participants receiving the facts only message demonstrated lower recall accuracy than all other message formats.	2
Cummings & Kong, 2019 [25]					Use of colloquial “flu shot” was more strongly associated with lower intention to vaccinate than formal “influenza vaccine.”			3
Herrett et al., 2015 [26]						In the standard care arm, mean vaccine uptake across practices was 50.7% and in the intervention arm uptake was 52.4% (not significant). There was a non-significant increase in vaccine uptake among at-risk patients aged 18–64 years in the intervention group, compared to standard care		4
Kim et al., 2019 [27]			Individuals exposed to both benefits and risk disclosure of the influenza vaccine tended to report lower perceived vaccine efficacy, which further significantly impacted felt ambivalence toward vaccination, and subsequently vaccination intention.		Individuals had higher vaccine intentions when only benefits of influenza vaccines were presented, excluding risk disclosure			2
Lee et al., 2018 [28]	Public service announcements (PSAs) pairing a gain-framed message with an image and a loss-framed message with text had positive effects on participants’ affect toward the PSAs	PSAs pairing a gain-framed message with an image and a loss-framed message with text had positive effects on participants’ attitude toward the influenza vaccine	PSAs pairing a gain-framed message with an image and a loss-framed message with text had positive effects on participants’ confidence in the influenza vaccine		PSAs that paired a gain-framed message with an image and a loss-framed message with text produced the most positive effects on participants’ vaccination intentions.			3
Macdonald et al., 2013 [29]						Three interventions aiming to increase uptake in Healthcare workers reported evidence of effectiveness. Changes in vaccination rates of 23.7% to 37% over 2 years [57] and differences in percentage uptake between a group receiving mixed-media messages (25.0%) compared to the control (16.0%) [58] was demonstrated. Two interventions targeting older adults reported evidence of effectiveness: increase from 45.0% 5 years previously to 70% after a three-year multi-faceted campaign [59], and increase from 21.7% to 51.7% after an intervention involving printed information at primary care clinic reception desks [60].		9 ꝉ
Prati et al., 2012 [30]	Narrative communication message was rated as more believable		Narrative message was related to higher perception of the efficacy of the vaccine	Narrative message was related to vaccination self-efficacy	No differences among the three conditions for vaccination intentions		Participants in the narrative communication condition reported a higher level of comprehension	4
Regan et al., 2017 [31]						12% of the intervention group and 9% of the control group were vaccinated. SMS reminder group were 39% more likely than the control group to receive a seasonal influenza vaccine. Shorter no. of days between the start of the study and vaccination uptake for the intervention group. Parents of high risk children were more likely to get their children vaccinated, 8.8% in the text message group compared to 3.6% in the control group		4
Yu & Shen, 2013 [32]	A gains framed message with individualistic appeal was perceived as more effective in both US & Hong Kong Chinese participants	Significantly more favourable attitude towards vaccination was reported by US participants when the messages were loss-framed with collectivistic appeal			Significantly higher intentions to get a vaccination was observed in both participant groups when the message was loss-framed and collectivistic			1
Chai et al., 2013 [33]						H1N1 SMS group had 1.77 times greater odds of receiving the new vaccine		3
Chien et al., 2011 [34]	A loss-framed message with white text on a red background was considered more reliable and prominent than the loss-framed message on a blue background.				Significantly higher willingness when a loss-framed message was presented with white text on a red background than when the message used white text on a blue background.			2
Driedger et al., 2013 [35]	Participants found language used to describe priority groups ‘at risk’ was discriminatory. Some felt there was a conspiracy against Native people.							5
Jhummon-Mahadnac et al., 2012 [36]			54.8% of participants believed seasonal influenza vaccine does not protect against pandemic (H1N1) influenza, 23.8% were unsure.		14.3% had vaccination intentions. Reasons for not intending to get the vaccine included perceiving self to be at low risk (30.5%); Vaccine has side effects (19.5%); Could not be bothered (17.5%). New vaccine may not be effective next year due to viral changes (15.6%) & prepared to wait for winter (11.7%)	22.2% of participants reportedly received the new vaccine.		5
Lin et al., 2014 [37]						Those who felt official authorities had openly provided the public with clear and honest information about pandemic influenza vaccination believed they were sufficiently informed and were more likely to get immunized.		10 ꝉ
Lynch et al., 2012 [38]					Majority of participants expressed some uncertainty about whether to get vaccinated while pregnant. 48.5% reported vaccination intentions		Unfamiliarity with antiviral medicine and terminology influenced vaccine acceptability. Some participants were concerned about potential side effects of vaccine on the fetus.	5
Miczo et al., 2013 [39]							The most frequently mentioned messages students remembered were: to wash hands (56.9%), self-isolation (23.5%) and getting a vaccination (22.1%).	2
Nan et al., 2012 [40]			Higher perceived vaccine efficacy was associated with more favorable attitudes toward H1N1 vaccination		When perceived vaccine efficacy was low the loss-framed message was significantly more effective than the gain-framed message in improving vaccination intentions			4
Ou et al., 2014 [41]	Attitude to medical information was influenced by perceptions that the message was informative and the message was credible.							0
Prati et al., 2011 [42]						2.8% of respondents reported receiving the vaccine.		3
Teasdale & Yardley 2011 [43]		A common perceived barrier to vaccination was safety concerns due to doubts about the testing of the vaccine during the expedited development						4
Godinho et al., 2016 [52]	The shortened Department of Health (DoH) message was rated as being more personally relevant, despite being considered as slightly less credible than the longer version. Those receiving the ‘shortened risk-reducing’ message rated the message as being clearer when compared to either those receiving the ‘shortened health-enhancing’ or the shortened DoH message.		Those receiving the ‘shortened risk-reducing’ message perceived vaccination to be more beneficial compared to either those receiving the ‘shortened health-enhancing’ or the shortened DoH message		Participants in the Standard DoH message condition showed lower vaccination intentions compared with the Shortened DoH message condition. The effect of message length on intention was explained by increase in perceived susceptibility and anticipated regret, the lowering of perceived costs of vaccination, increased perceived relevance of the information and message readability.		The information presented in the shortened DoH message was better recalled when compared to the other two conditions.	5
Han et al., 2018 [53]			The Uncertainty group demonstrated significantly lower perceived vaccine effectiveness		The Uncertainty group showed significantly lower vaccination interest than the No-Uncertainty group. As health literacy increased, the difference in vaccination interest between uncertainty groups and the No-Uncertainty group increased, demonstrating greater ambiguity aversion for higher-literate individuals and greater ambiguity tolerance for lower-literate individuals			5
Kononova et al., 2016 [54]					When multitasking with Facebook, individuals indicated greater intentions to follow vaccine recommendations			4
Mowbray et al., 2016 [55]	Factual, evidence-based messages were found to be the most convincing and useful and were well received. Health-enhancing messages were received with scepticism. Risk-reduction messages were perceived as being more balanced and credible.	Concern about messages not being honest and about the potential lack of safety.						4
Fitzpatrick-Lewis et al., 2010 [56]			The relative risk format resulted in higher ratings of perceived effectiveness of vaccination than the absolute format. Baseline information about risk led to higher ratings of perceived effectiveness of vaccination.		The relative risk format resulted in higher vaccination intentions than the absolute format. Baseline information about risk led to higher ratings of likelihood of being vaccinated			9 ꝉ
Lapka et al., 2008 [48]			Even after reading the messages, most participants still believed the influenza vaccine causes the flu.				Many participants could not describe how long it takes the vaccine to build immunity and were confused about the possibility of getting influenza during the two weeks following vaccination.	5
Mayweg-Paus & Jucks, 2015 [49]		Participants who had received hints about the source reported fewer positive statements about vaccination than participants receiving no hints.						1
Payaprom et al., 2011 [50]				Participants in the intervention group showed a greater increase in self-efficacy from Time 1 to Time 2 than control group participants	Participants in the intervention group showed a greater increase in vaccination intentions from Time 1 to Time 2 than control group participants	Vaccination uptake did not differ between groups	Significant increases in knowledge of vaccine side effects in both Health Action Process Approach (HAPA) leaflet and standard leaflet groups between Time 1 and Time 2	3
Phillips et al., 2014 [51]						Vaccination rates were 49.3% in the intervention group versus 46.6% in the usual care group After adjusting for gestational age and number of clinic visits, women who received text messages were more likely to receive an influenza vaccination		1 ꝉ
Shenson et al., 2001 [45]						Increased uptake in vaccinations in the target county vs the non-target county. Increased uptake in the target county after messages compared to uptake in the two years prior to implementation		2
Ort & Fahr, 2018 [47]		Negative influence of perceived threats on attitudes in favour of the proposed vaccination was found	Positive relationship between perceived efficacy and a more favourable vaccination attitude		Positive relationship between an attitude in favour of the vaccination and vaccination intentions			3
Wolf et al., 2015 [44]		Participants who viewed images of children with measles were more likely to associate autism with vaccines			Parents reported increased vaccine intentions when additional information emphasised vaccine benefits directly for the child or both to the child and society			1 ꝉ
Kelly & Hornik, 2016 [46]					The “society” condition resulted in significantly higher intentions than the “self” condition			3

ꝉ AMSTAR was used to assess quality.

**Table 2:** Summary of moderate to high quality evidence of methods to support vaccination uptake, beliefs and intentions with supporting evidence from a broader review of public health messaging [16].

Methods to Support Acceptable Messages	Evidence	Quality of Evidence	Supporting Evidence
Uptake			
Community-wide mixed media campaigns found to be effective for improving vaccination uptake among older adults.	[29,45] (systematic review)	Moderate High	Use different media for delivery and match delivery to the population’s needs and perceptions [16]
Hospital-wide mixed media campaigns including educational and advertising methods sent to healthcare workers improved vaccination uptake	[29] (systematic review)	High	
Text messages including information about health risks, vaccine safety and recommending vaccination were effective for increasing vaccine uptake among pregnant women.	[23]	High	Increase the public’s awareness of the risks of the virus to their own health and the health of others [16]
Community-wide text message prompts with information about virus prevention and addressing misunderstandings about vaccination increased vaccination uptake among the general population	[33]	Moderate	Identify inconsistencies in messages from uncontrolled sources, especially when addressing key preventative behaviours [16]
Including a map with the locations of influenza vaccination clinics in email invitations for vaccinations increased vaccination uptake.	[22]	Moderate	Frame the message to emphasise positive beliefs about one’s own health and that preventative behaviour is within one’s control [16]
Psychological influences over uptake			
Shortened messages from official sources that were personally relevant, included information about susceptibility, and were risk-reducing were more effective than longer messages for improving willingness to be vaccinated.	[52]	High	Deliver consistent, clear, core messages about risk and preventative behaviour across sources within the same time points [16]
Fear of side effects, concerns about risks to unborn baby, and unfamiliarity with vaccine terminology in messages were found among pregnant women, impacting on vaccination intentions.	[38]	High	Tailor key messages to be applicable to an individual’s situation [16]
Vaccine safety concerns may arise from messages about the speed new vaccines have been tested during pandemics and have impacted on willingness to be vaccinated.	[43]	High	Be transparent: admit errors and unknowns whenever appropriate [16]
Messages focused on benefits to society were found to be more effective than messages emphasising benefits to the self, to increase vaccination intentions.	[46]	Moderate	Consider framing messages around social responsibility and norms [16]
A leaflet including influenza susceptibility, severity, vaccination benefits and efficacy, and behaviour change techniques including providing information about the behaviour-health link and personal accounts of people who received vaccination increased vaccination intentions	[50]	Moderate	Increase the public’s awareness of the risks of the virus to their own health and the health of others [16]
Factual, risk-reducing messages were perceived as more credible and resulted in beliefs vaccination is more beneficial than messages emphasising health benefits of vaccines.	[52,55]	High High	Increase factual knowledge of all aspects of a virus (e.g., symptoms) and benefits of preventative behaviour using an appropriate message frame [16]
Providing baseline information about risk alongside relative risk framing to communicate risk can result in stronger beliefs about effectiveness than using absolute risk framing.	[56] (systematic review)	High	Accurately describe the health threat, severity of the threat, the risk to self and others, coupled with information about how to reduce the risk [16]
Narrative messages targeting confidence in vaccines, including stories of adults over 65 affected by seasonal influenza who got vaccinated, improved beliefs about capability to take up a vaccine.	[30]	High	Tailor key messages to be applicable to an individual’s situation [16]
Pairing images of young adults while emphasising gains associated with vaccination, or framing losses with text (i.e., avoiding the use of negative imagery) increased confidence in vaccination effectiveness among young adults	[28]	Moderate	Increase factual knowledge of all aspects of a virus (e.g., symptoms) and benefits of preventative behaviour using an appropriate message frame [16]
Web pages describing general information about Ebola and efforts involved in developing a vaccine and providing strong statements about self-efficacy and response efficacy were linked to more favourable attitudes towards vaccination.	[47]	Moderate	Accurately describe the health threat, severity of the threat, the risk to self and others, coupled with information about how to reduce the risk [16]
Acceptability of messages & information needs			
A lack of clarity in messages using vaccine-related terminology and scientific information (e.g., the time it takes to build immunity) can impact on message acceptability among high risk groups	[38,48]	High High	Engage with key stakeholders and communities [16]
High risk groups may perceive priority to be vaccinated using new vaccines as a form of discrimination, impacting negatively on attitudes towards the message.	[35]	High	Use messaging that empowers communities to take control of their own health [16]
Factual, risk-reducing messages may be perceived as more credible than health-enhancing messages	[55]	High	Increase factual knowledge of all aspects of a virus (e.g., symptoms) and benefits of preventative behaviour using an appropriate message frame [16]
Messages from official sources challenging myths may be more effective than providing facts alone for improving knowledge about vaccines	[24]	Moderate	Identify inconsistencies in messages from uncontrolled sources, especially when addressing key preventative behaviours [16]

## Data Availability

Not applicable.

## Supplementary Materials

The following are available online at https://www.mdpi.com/2076-393X/9/2/72/s1, Table S1 PPI Checklist; Table S2 Characteristics of included studies.
