# Supplementary material for: A Rapid Systematic Review of Public Responses to Health Messages Encouraging Vaccination against Infectious Diseases in a Pandemic or Epidemic
Source: Vaccines (Basel). 2021 Jan 20;9(2):72. doi: 10.3390/vaccines9020072 (PMC7909419; doi:10.3390/vaccines9020072)
Supplement: Supplementary file 1 [file vaccines-09-00072-s001.pdf]

Table S1. PPI Reporting in Studies Checklist

	Type of PPI	N/A	No	Unclear	Yes
**Journal**	Journal has a PPI reporting policy				
**Study design**	Contributions to the grant application and or study protocol*				
	Ensuring the development of, or choice of, outcome measures were informed by patients' priorities, experience and preferences*				
	Patient assessment of the burden of the intervention before the study commenced or involvement in designing the intervention (where applicable)*				
**Study conduct**	Involved in the study steering group or a member of the research team*				
	Recruitment and/or implementation of the research*				
	Patient/public communication materials e.g., patient info sheets*				
**Analysis**	Contributed to data analysis				
	Interpretation of study findings				
**Drafting of manuscript**	Contributions to editing, revising or writing the manuscript				
	Patients listed as co-authors				
**Dissemination**	Direct involvement of patients led by the research team including the development of materials for dissemination and choosing the most appropriate method of dissemination				

	Indirect involvement through dissemination to patient charities, organisations and groups that may, in turn, involve patients in the process				
	Patient representation informing the content of dissemination materials on a general advisory board for the use of the data used in research				
Other	Any other PPI involvement				

* *Adapted from Price, A., et al., Frequency of reporting on patient and public involvement (PPI) in research studies published in a general medical journal: a descriptive study. BMJ open, 2018.* ***8****(3)*

Table S2. Characteristics of included studies

Authors, year, country	Study Design	Sample size (n)	Infectious disease	Time point[ł] in crisis	Description of messaging intervention	Outcomes measured				PPI
						Perceptions	Intentions	Behaviour	Other	
**Baskin, 2018, US [22]**	Cross sectional, quantitative, randomised	University students, faculty and staff (n = 30,748)	*Seasonal influenza*	Pre & peak	The influenza vaccination program included an email sent out to all members of the university to announce the dates, times, and locations of vaccination clinics. The university followed up with messages to targeted segments of the population based on age and other risk factors.<br><br>12 conditions were used: Map Inclusion: Yes or No x Negative Impacts of Reminder: None or sickness reminder or work reminder x Incentive: Yes or No			Vaccination uptake		None
**Bushar et al, 2017, US [23]**	Cross sectional, quantitative, non-randomised	Pregnant women (n = 3,321)		Pre	Free mobile health (mHealth) service "Text4baby" sending three weekly texts educating women about important health issues, encouraging contact with providers, and promoting healthy behaviors including influenza vaccine.			Vaccination uptake		None

Authors, year, country	Study Design	Sample size (n)	Infectious disease	Time point[ǂ] in crisis	Description of messaging intervention	Outcomes measured				PPI
						Perceptions	Intentions	Behaviour	Other	
**Cameron et al, 2013, US [24]**	Pretest-posttest, quantitative, randomised	Adults over 50 years (n = 125)		Pre & peak	Printed message flyers with four different influenza related messages:<br>1. Facts Only<br>2. Facts and Myths<br>3. Facts, Myths, and Refutations<br>4. CDC Control (providing a myth and facts)				Knowledge about vaccination<br><br>Information recall	Public were involved in piloting messages (but for manipulation purposes only)
**Cummings & Kong, 2019, Singapore [25]**	Cross-sectional, quantitative, randomised	General population (n=896)		NR	Questionnaires asked about influenza vaccination intentions used either "influenza vaccine" or "flu shot" in the wording		Intentions to take up vaccine			None
**Herrett et al, 2015, UK [26]**	Clustered RCT	Adults with chronic conditions (n= 102,257)		During	Two messaging conditions:<br>1. Standard care involved practices continuing with their influenza vaccination campaign, typically using posters in the practice and letters to patients.<br>2. Practices in the intervention arm additionally sent a text message vaccination reminder using their in-			Vaccination uptake		None

Authors, year, country	Study Design	Sample size (n)	Infectious disease	Time point[ǀ] in crisis	Description of messaging intervention	Outcomes measured				PPI
						Perceptions	Intentions	Behaviour	Other	
					practice text messaging software, to patients aged 18–64 years in at-risk groups. The text message mentioned vaccine reduces risk of serious health problems from flu.					
**Kim et al, 2019, US [27]**	Cross-sectional, quantitative, randomised	University students (n=86)		NR	Health information flyer with Department of Public Health (DPH) branding provided brief information about influenza. There were two message conditions:<br>1. Gain-framed with risk disclosure message presented positive outcomes of flu vaccination and vaccine side effects.<br>2. Gain-framed-only message included the positive outcomes of flu vaccination and the benefits of vaccination	Ambivalence towards vaccination<br><br>Perceived vaccine efficacy	Intentions to take up vaccine			None
**Lee et al, 2018, US [28]**	Cross-sectional, quantitative, randomised	University students (n=122)		NR	Public service advertisements (PSAs) with four different messages:<br>1. A loss frame with image support	Attitude toward flu vaccine	Intentions to take up vaccine		Attitude toward vaccine PSAs	None

Authors, year, country	Study Design	Sample size (n)	Infectious disease	Time point[ł] in crisis	Description of messaging intervention	Outcomes measured				PPI
						Perceptions	Intentions	Behaviour	Other	
					2. A gain frame with image support<br>3. A loss frame with text support<br>4. A gain frame with text support	Confidence in safety of flu vaccine				
**Macdonald et al, 2013, UK [29]**	Systematic review	NR		Various	- Mass communication (distribution of universally targeted information to undifferentiated or large segments the population at the same time).<br>- Personalized communication (which aims to make a personally relevant appeal to individuals by, for example, using direct contact or individually addressed correspondence). Four studies evaluated verbal prompts from healthcare professionals to encourage vaccination (not messages eligible for our review).<br>- Training/education.		Intentions to take up vaccine	Vaccination uptake		Not reported in review
**Prati et al, 2012, Italy [30]**	Cross-sectional, quantitative, randomised	Adults over 65 years (n = 311)		NR	Messages looked like a mass-media communication campaign carried out by the Italian Minister of Health.	Perceived vaccine efficacy	Intentions to take up vaccine		Understanding of the message	Public were involved in

Authors, year, country	Study Design	Sample size (n)	Infectious disease	Time point[†] in crisis	Description of messaging intervention	Outcomes measured				PPI
						Perceptions	Intentions	Behaviour	Other	
					Didatic vs narrative messages were used. Narrative messages featured stories of people aged 65 and over touched by seasonal influenza and who got the vaccine.	Believability of the message<br><br>Vaccination self-efficacy				piloting messages (but for manipulation purposes only)
**Regan et al, 2017, Australia [31]**	RCT	Adults at high risk of severe influenza (n = 12,354)		Pre	SMS messages read: This is a message from <<PRACTICE NAME>> for <<FIRST NAME>>. Flu season is approaching. You may be eligible for government-funded influenza vaccine and our records show you have not yet been vaccinated. Please call <<PRACTICE PHONE>> if you would like to schedule an appointment.			Vaccination uptake		None
**Yu & Shen, 2013, US & Hong Kong [32]**	Cross-sectional, quantitative, randomised	University students:<br>• US = 126<br>• Hong Kong = 116		Pre	Culturally-tailored (collectivistic vs individualistic) messages in printed brochures with information about the risk of influenza, a quote from a doctor, and basic information about	Attitudes towards vaccination	Intentions to take up vaccine		Attitudes towards message	None

Authors, year, country	Study Design	Sample size (n)	Infectious disease	Time point[ł] in crisis	Description of messaging intervention	Outcomes measured				PPI
						Perceptions	Intentions	Behaviour	Other	
					vaccination. Four different brochures were tested:<br>1. A loss-frame with an individualistic appeal: Skipping a Flu Shot May Put You at Risk<br>2. A loss-frame with a collectivistic appeal: Skipping a Flu Shot May Put Many at Risk<br>3. A gain-frame with an individualistic appeal: Getting a Flu Shot May Benefit You<br>4. A gain-frame with a collectivistic appeal: Getting a Flu Shot May Benefit Many					
**Chai et al, 2013, China [33]**	RCT	General public (n = 1992)	*H1N1 pandemic*	Post	Two messaging conditions:<br>1. The intervention group received SMS messages about 2009 H1N1 prevention and control. Ten messages in total were sent over 10 days, two of which related to vaccination (one SMS recommended vaccination and another challenged			Self-reported vaccination uptake		None

Authors, year, country	Study Design	Sample size (n)	Infectious disease	Time point[ł] in crisis	Description of messaging intervention	Outcomes measured				PPI
						Perceptions	Intentions	Behaviour	Other	
					misconceptions the vaccine spread H1N1 flu).<br>2. The control group received messages about tobacco cessation.					
**Chien et al, 2011, Taiwan [34]**	Cross sectional, quantitative, non-randomised	University students (n=120)		NR	Information about vaccination adapted from CDC materials were delivered by television broadcast. Four messages were used:<br>1. Gain-framed white text on red background<br>2. Gain-framed white text on blue background<br>3. Loss-framed white text on red background<br>4. Loss-framed white text on blue background		Willingness to take up vaccine		Attitudes towards message	None
**Driedger et al, 2013, Canada [35]**	Qualitative (Focus group)	Aboriginal First Nations and Metis adults (n = 193)		During	Public messaging campaigns targeted towards the Aboriginal population (i.e., through the Aboriginal People's Television Network, translated radio announcements in local dialects, and a special information campaign through the Manitoba Metis	Attitudes towards vaccine			Attitudes towards messages	Public involved in identifying priorities and designing unspecif

Authors, year, country	Study Design	Sample size (n)	Infectious disease	Time point[ł] in crisis	Description of messaging intervention	Outcomes measured				PPI
						Perceptions	Intentions	Behaviour	Other	
					Federation to its citizen membership)					ied aspects
**Jhummon-Mahadnac et al, 2012, Australia [36]**	Cross-sectional, quantitative, non-randomised	Emergency dept patients, and visitors, non-clinical staff (n = 252)		During	Public Education Campaigns delivered via radio, written press and television promotions, federal government pandemic website, the Victorian Department of Human Services (DHS) website, and twitter feeds.	Perceived vaccine efficacy	Intentions to take up vaccine	Self-reported vaccine uptake		None
**Lin et al, 2014, US [37]**	Systematic review	NR		Various	Variety of interventions included, including 'Websites', ' Commercial television', 'Health department' (not specified in more detail in the review)	Perceived vaccine efficacy		Vaccination uptake		
**Lynch et al, 2012, US [38]**	Cross-sectional, qualitative (focus groups)	Pregnant and recently pregnant women (n = 144)		During	Video clips of news broadcasts focused on the April 2009 H1N1 outbreak		Intentions to take up vaccine		Information needs about vaccine	None
**Miczo et al, 2013, US [39]**	Cross-sectional, quantitative, non-randomised	University students (n=204)		Post	Campaigns on campus (flyers, posters, emails). Mass media (various, including television & radio) other sources (e.g. medical) were disseminated recommending a range of				Information recall	None

Authors, year, country	Study Design	Sample size (n)	Infectious disease	Time point[ł] in crisis	Description of messaging intervention	Outcomes measured				PPI
						Perceptions	Intentions	Behaviour	Other	
					protective behaviours, including hand washing, using sanitizer and getting a vaccine.					
**Nan et al, 2012, US [40]**	Cross sectional, quantitative, randomised	Adults over 50 years (n=88)		Post	A short message from the CDC explaining influenza, health problems caused by it, and the availability of the 2010–2011 seasonal influenza vaccine (including vaccines against seasonal flu and the H1N1 flu).<br><br>A further message was presented in a health pamphlet that was either:<br>1. Gain-framed focused on the benefits of receiving the H1N1 flu vaccine<br>2. Loss-framed emphasizing the costs of not receiving the H1N1 flu vaccine	Attitudes towards vaccine<br><br>Perceived vaccine efficacy	Intentions to take up vaccine			None
**Ou et al, 2014, Taiwan [41]**	Cross-sectional, quantitative, non-randomised	Surgical inpatients (n = 463)		Post	Government-delivered risk communication information.	Attitudes towards message				None

Authors, year, country	Study Design	Sample size (n)	Infectious disease	Time point[ł] in crisis	Description of messaging intervention	Outcomes measured				PPI
						Perceptions	Intentions	Behaviour	Other	
**Prati et al, 2011, Italy [42]**	Cross-sectional, quantitative, non-randomised	General public (n=1010)		During	Social and educational campaigns delivered by TV broadcast recommended behaviours for the public including using tissues when sneezing, washing hands regularly with soap and water, cleaning or disinfecting objects that one might touch, social distancing and vaccine acceptance			Self-reported vaccine uptake		None
**Teasdale & Yardley 2011, UK [43]**	Cross-sectional, qualitative (Focus groups)	Parents, teachers, college students & university staff (n = 48)		During	Participants were presented with government advice on recommended actions. Participants were presented with a hypothetical high threat and low threat scenario to discuss in the group.	Perceived effectiveness of vaccine<br><br>Confidence in safety of flu vaccine				None
**Godinho et al, 2016, UK [52]**	Cross-sectional, quantitative, randomised	General public (n=1424)	*Hypothetical influenza pandemic*	N/A	A mock newspaper article about a novel influenza describing uncertainty about the pandemic, in one of four conditions:<br>1. The standard Department of Health (DoH) message adapted from the real leaflet used in the 2009–10 "swine flu" pandemic.	Attitude towards the message<br><br>Perceived vaccine efficacy	Intentions to be vaccinated		Information recall	None

Authors, year, country	Study Design	Sample size (n)	Infectious disease	Time point[ł] in crisis	Description of messaging intervention	Outcomes measured				PPI
						Perceptions	Intentions	Behaviour	Other	
					2. A shortened DoH message contained key information selected from the leaflet and targeted known vaccination predictors: knowledge about flu and precautionary measures, perceived susceptibility, perceived costs (emphasising low risk of side effects and vaccine safety) and vaccine efficacy 3. A shortened risk-reducing message presented the vaccine as a way of reducing the risk of contracting pandemic flu. The severity of the pandemic influenza was emphasised. 4. A shortened health-enhancing message presented the vaccine as a way of boosting the immune system and maintaining good health, and emphasised the severity of pandemic influenza.					
**Han et al, 2018, US [53]**	Cross sectional,	Spanish adults (n = 2701)		N/A	Vignettes of hypothetical newspaper articles. Participants were instructed	Perceived vaccine efficacy	Intentions to take up vaccine			None

Authors, year, country	Study Design	Sample size (n)	Infectious disease	Time point[ł] in crisis	Description of messaging intervention	Outcomes measured				PPI
						Perceptions	Intentions	Behaviour	Other	
	quantitative, randomised				to imagine there has been an outbreak of the flu, that cases were rising, and that health officials are concerned. Three message conditions included:<br>1. No uncertainty: Participants were informed health officials were confident the outbreak would be bad<br>2. Uncertainty: Participants were informed it was too soon to tell how severe the outbreak would be<br>3. Normalised uncertainty: Emphasised that uncertainty was normal					
**Kononova et al, 2016, US [54]**	Cross sectional, quantitative, randomised	University students (n = 121)		N/A	Two messaging conditions:<br>1. Multitasking condition: Participants read an online article about the flu on a health-related website and checked Facebook while reading<br>2. Control: Participants read the same article without interruptions		Intentions to take up vaccine			None

Authors, year, country	Study Design	Sample size (n)	Infectious disease	Time point[ł] in crisis	Description of messaging intervention	Outcomes measured				PPI
						Perceptions	Intentions	Behaviour	Other	
**Mowbray et al, 2016, UK [55]**	Cross sectional, qualitative (Focus Groups)	General public (n = 41)		During	Participants were presented with a brief, hypothetical scenario describing what might happen during a pandemic influenza outbreak, including information on health consequences, impact and vaccination advice	Attitudes towards vaccine				None
**Fitzpatrick-Lewis et al, 2010, Canada [56]**	Systematic review	General public (n = 220)		N/A	Natter et al (2005) - The fictitious scenario informed participants about a severe influenza epidemic that was expected<br><br>Half of the participants in both risk reduction formats were informed about baseline risk: ‘‘It is predicted that 10% of the adult population (i.e., 10 out of every 100 adults) will be affected by the flu’’. The scenario advised people they should be vaccinated.<br><br>The absolute risk reduction was communicated as: ‘‘With vaccination, the risk	Perceived vaccine efficacy	Intentions to take up vaccine			None

Authors, year, country	Study Design	Sample size (n)	Infectious disease	Time point[ł] in crisis	Description of messaging intervention	Outcomes measured				PPI
						Perceptions	Intentions	Behaviour	Other	
					of being affected by the flu is 5% lower''.<br><br>The wording of the relative risk reduction was: ''With vaccination, the risk of being affected by the flu is reduced by 50%''.					
**Lapka et al, 2008, US [48]**	Cross-sectional, qualitative	African American adults over 50 years (n = 10)	*Unspecified influenza*	NR	Testing comprehension of four flu vaccination topics: "The Flu Shot Doesn't Give You the Flu", "The Flu Shot is Safe", "The Flu Shot Works" and "Benefits and Risks". Participants were shown nine sections with text excerpts from each of the four message topics.	Perceived vaccine efficacy			Understanding of message	None
**Mayweg-Paus & Jucks, 2015, Germany [49]**	Cross-sectional, mixed, non-randomised	Students in final year of higher secondary education (n = 157)		NR	A short journalistic text in a magazine, briefly introducing a new vaccine. Four different versions of the text manipulated: the presence or absence of lexical hedges (e.g. "possibly, "potentially"), and the presence of absence of hints on information source (e.g. "The researchers	Attitudes towards vaccination				None

Authors, year, country	Study Design	Sample size (n)	Infectious disease	Time point[ł] in crisis	Description of messaging intervention	Outcomes measured				PPI
						Perceptions	Intentions	Behaviour	Other	
					report", "according to experts").					
**Payaprom et al, 2011, Thailand [50]**	Longitudinal, controlled before-after trial	Adults 45-65 years with one or more chronic conditions (n=201)		NR	Two leaflets were compared.<br>1. Health Action Process Approach (HAPA) was used to highlight susceptibility to influenza and its complications and the benefits of vaccination. The leaflet also included personal accounts of people who had received the vaccination and prompted specific goal setting.<br>2. Standard government information describing symptoms of influenza, details about the flu vaccine, possible side effects, and the general benefits of influenza vaccination	Vaccination self-efficacy	Intentions to take up vaccine	Vaccination uptake	Knowledge about vaccine side effects	None
**Phillips et al, 2014, US [51]**	Editorial review	Pregnant women (n=1187)		Early	Stockwell et al (2014) RCT - Women in the intervention group received a sequence of 5 weekly, automated text messages including influenza vaccine reminders.			Vaccination uptake		None

Authors, year, country	Study Design	Sample size (n)	Infectious disease	Time point[ǂ] in crisis	Description of messaging intervention	Outcomes measured				PPI
						Perceptions	Intentions	Behaviour	Other	
					Messages provided information, (1) about increased risks of influenza-related illness among pregnant women and babies, (2) vaccine safety, and (3) that doctors recommend the influenza vaccine. A further message enabled women to select more information about influenza risk, common misperceptions about vaccines, side effects, and need for yearly influenza vaccination					
**Shenson et al, 2001, US [45]**	Cross sectional, quantitative, non-randomised epidemiological analysis	Medicare beneficiaries in Duchess County, New York (n = 7,961)	*Pneumococcal*	NR	A community-wide outreach campaign promoting pneumococcal and influenza immunizations. Brochures, letter from well known health care leaders, radio show, radio advertisement, and newspaper advertisements reported details for flu clinics and reasons for getting vaccinated.			Vaccination uptake		Public were involved in the intervention design and in a steering group
**Ort & Fahr, 2018,**	Cross-sectional,	University students (n = 447)	*Ebola virus*	During	A mock website containing information about Ebola, the efforts involved in	Attitudes towards vaccination	Intentions to take up vaccine			None

Authors, year, country	Study Design	Sample size (n)	Infectious disease	Time point[ł] in crisis	Description of messaging intervention	Outcomes measured				PPI
						Perceptions	Intentions	Behaviour	Other	
**Switzerland [47]**	quantitative, randomised				developing a vaccination, efficacy–related (protective vaccination) information and a recommendation to get vaccinated. Four message conditions were used:<br>1. Threat low – efficacy low<br>2. Threat low – efficacy high<br>3. Threat high – efficacy low<br>4. Threat high – efficacy high	Vaccination self-efficacy				
**Wolf et al, 2015, US [44]**	Editorial review	NR	*MMR epidemics*	Various	Nyhan et al 2014: Participants received a placebo message or one of the following 4 messages:<br>1. A message that dispelled MMR vaccination myths,<br>2. A message providing textual information explaining the risks of measles,<br>3. A message including images of children with measles and<br>4. A message including a dramatic narrative.<br><br>Hendrix et al 2014<br>One of 4 messages was sent to parents of infants:	Concerns about vaccine side effects	Intentions to take up vaccine			Not reported in review

Authors, year, country	Study Design	Sample size (n)	Infectious disease	Time point[ł] in crisis	Description of messaging intervention	Outcomes measured				PPI
						Perceptions	Intentions	Behaviour	Other	
					1. CDC and Prevention Vaccine Information Statement (VIS),<br>2. VIS and information emphasizing the MMR vaccine's benefits to the child,<br>3. VIS and information emphasizing societal benefits,<br>4. VIS and information emphasizing benefits both to the child and society					
**Kelly & Hornik, 2016, US [46]**	Cross-sectional, quantitative, randomised	General public (n=485)	*Avian influenza*	Pre	The authors based messages on WHO and CDC avian flu information at the time of the study. All were gain-framed, i.e. what might be gained from vaccination. All messages were phrased in hypothetical terms (e.g., "when a vaccine becomes available").<br><br>Participants were randomized to receive either:<br>1. A self-focused message stating some might not get ill, but some would		Intentions to take up vaccine			Public were involved in piloting messages (but for manipulation purposes only)

Authors, year, country	Study Design	Sample size (n)	Infectious disease	Time point[ł] in crisis	Description of messaging intervention	Outcomes measured				PPI
						Perception s	Intentions	Behaviour	Other	
					2. A close other message emphasising even if a person did not get ill, he/she could be a carrier and pass the illness to "spouse, children and other family members"<br>3. The society only message mentioned "other" people instead of loved ones.					
